# COVID-19–Related School Closures and Learning Modality Changes — United States, August 1–September 17, 2021

**DOI:** 10.15585/mmwr.mm7039e2

**Published:** 2021-10-01

**Authors:** Sharyn E. Parks, Nicole Zviedrite, Samantha E. Budzyn, Mark J. Panaggio, Emma Raible, Marc Papazian, Jake Magid, Faruque Ahmed, Amra Uzicanin, Lisa C. Barrios

**Affiliations:** ^1^CDC COVID-19 Response Team; ^2^Booz Allen Hamilton, McLean, Virginia; ^3^Johns Hopkins University Applied Physics Laboratory, Laurel, Maryland; ^4^Palantir Technologies, Denver, Colorado.

Beginning in January 2021, the U.S. government prioritized ensuring continuity of learning for all students during the COVID-19 pandemic (*1*). To estimate the extent of COVID-19–associated school disruptions, CDC and the Johns Hopkins University Applied Physics Laboratory used a Hidden Markov Model (HMM) (*2*) statistical approach to estimate the most likely actual learning modality based on patterns observed in past data, accounting for conflicting or missing information and systematic Internet searches (*3*) for COVID-19–related school closures. This information was used to assess how many U.S. schools were open, and in which learning modalities, during August 1–September 17, 2021. Learning modalities included 1) full in-person learning, 2) a hybrid of in-person and remote learning, and 3) full remote learning.

Multiple data sources were combined to estimate the learning modality for public and public charter school districts in the United States using HMM; sources included Burbio,* MCH Strategic Data,^†^ American Enterprise Institute–Return to Learn,^§^ and state dashboards.^¶^ Weekly learning modalities (full in-person, hybrid, and full remote) during August 1, 2020–July 31, 2021 were used to select the optimal weights for each reported modality in order to infer the most likely actual learning modality. The trained HMM was applied weekly during August 1–September 17, 2021. In addition to using HMM, since February 2020, CDC has also tracked district and individual public and private school closures attributed to COVID-19 and estimated the number of students and teachers affected by these closures. School closure data were obtained via daily systematic Internet searches, as described previously (*3*), which identified publicly announced COVID-19–related closures lasting ≥1 day. School or district closure was defined as a transition from being open to being closed for in-person instruction. Fully in-person and hybrid (i.e., latter includes both in-person and remote) learning modalities were classified as open; fully remote learning modalities (if stated as offered during closure) were classified as closed. Closure dates and reasons were recorded and linked to publicly available education data.** HMM was fitted using the Pomegranate module (version 0.14.3) for Python (version 3.7.6). COVID-SC data were imported into SAS (version 9.4; SAS Institute) for analysis. These activities were reviewed by CDC and were conducted consistent with applicable federal law and CDC policy.^††^

For the week ending September 17, 2021, HMM data were available for 73% of kindergarten through grade 12 public school students in 8,700 districts nationwide and varied by state (Supplementary Figure, https://stacks.cdc.gov/view/cdc/109969). Among these districts, 8,343 (96%) were offering full in-person learning, 322 (4%) were offering hybrid learning, and 35 (0.4%) were offering full remote learning. The largest number of districts with full remote learning (14) were in the West Census Region, followed by the South (11). Seven Midwest and two Northeast districts offered full remote learning. During August 2–September 17, 2021, systematic Internet searches identified announcements of 248 public districtwide closures and 384 individual school closures for ≥1 day attributable to COVID-19. Closures affected 1,801 schools (1.5% of all schools), 933,913 students, and 59,846 teachers in 44 states (Figure). The number of closures was highest in the South.

**FIGURE:**
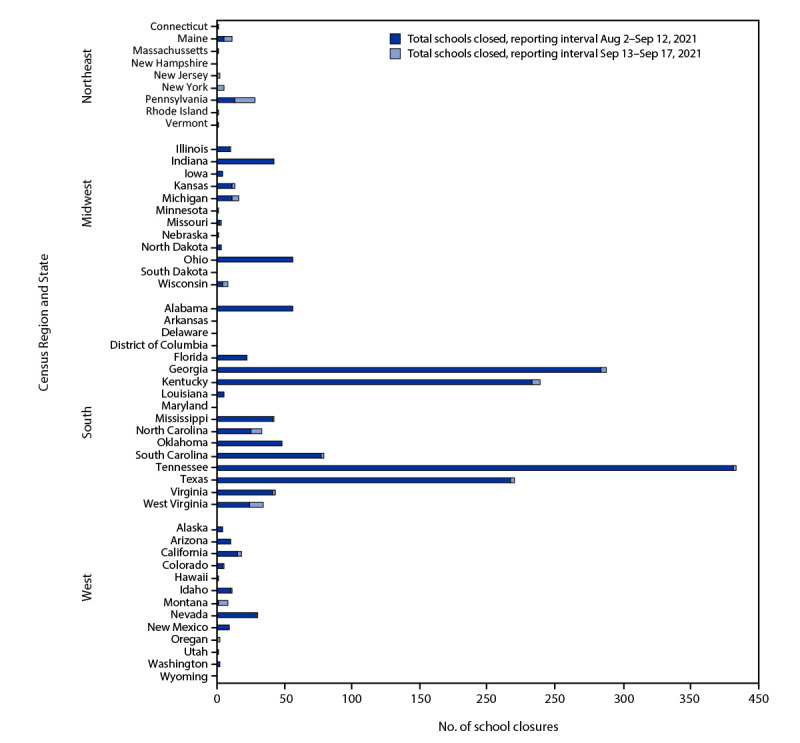
COVID-19–related kindergarten through grade 12 school closures, by region and state — United States, August 2–September 17, 2021

The findings in this report are subject to at least five limitations. First, both HMM and daily Internet searches were informed by passive collection of available information obtained through school and district surveys, public-facing website pages, and media reports; therefore, they are likely not inclusive of all school districts nationwide. Second, HMM did not account for the possibility of serial errors in sources (i.e., sources that are incorrect week after week). Third, districts included in HMM were larger than those excluded, thus limiting generalizability. Fourth, HMM is based on the assumption that probabilities for subsequent weeks are determined exclusively by the modality for the current week with no change in these probabilities over time or from district to district, both of which might not always be true. The results do not speak directly to level of impact because districts and schools may have different thresholds for closure or change in modality. Finally, regional differences must be interpreted cautiously. The timing of return to school likely accounts for some regional variation in school closures because longer in-session time increases opportunities for COVID-19 cases to appear in schools. Many districts in the South returned to school in early August compared with late August or early September return dates in other regions (*4*).

Federal public health and education agencies are using HMM model information and systematic Internet searches to identify districts and schools most affected by COVID-19–related disruptions. Examination of prevention activities in those with and without disruption can suggest modifications to COVID-19 prevention activities. CDC is currently making findings from these activities available to state and local public health and education agencies.

Most (96%) public and private schools have remained open for full in-person learning. However, an estimated 1,800 schools have had school closures attributable to COVID-19 outbreaks, affecting the education and well-being of 933,000 students. To prevent COVID-19 outbreaks in schools, CDC recommends multicomponent prevention strategies, including vaccination, universal indoor masking, screening testing, and physical distancing (*5*).

## Footnotes

*
https://cai.burbio.com/school-opening-tracker/

†
https://www.mchdata.com/covid19/schoolclosings

§
https://www.returntolearntracker.net/

¶ Colorado, Connecticut, Hawaii, Idaho, Illinois, Louisiana, Minnesota, Missouri, New Mexico, North Carolina, Ohio, Oregon, South Carolina, Tennessee, Vermont, Virginia, and Washington.

**
https://nces.ed.gov/ccd/districtsearch/

†† 45 C.F.R. part 46, 21 C.F.R. part 56; 42 U.S.C. Sect. 241(d); 5 U.S.C. Sect. 552a; 44 U.S.C. Sect. 3501 et seq.
